# Cell shape: effects on gene expression and signaling

**DOI:** 10.1007/s12551-020-00722-4

**Published:** 2020-07-15

**Authors:** Payam Haftbaradaran Esfahani, Ralph Knöll

**Affiliations:** 1 ICMC (Integrated Cardio Metabolic Centre), Myocardial Genetics, Heart and Vascular Theme, Karolinska Institutet, University Hospital, Novum, Hiss A, våning 7, Hälsovägen 7-9, 141 57 Huddinge, Sweden; 2 Bioscience Cardiovascular, Research and Early Development, Cardiovascular, Renal and Metabolism (CVRM), BioPharmaceuticals R&D, AstraZeneca, Gothenburg, Sweden

**Keywords:** Cell geometry,, Cell shape,, Gene expression,, Heart failure,, Mechanosensation,, Mechanotransduction

## Abstract

The perception of biophysical forces (mechanosensation) and their conversion into chemical signals (mechanotransduction) are fundamental biological processes. They are connected to hypertrophic and atrophic cellular responses, and defects in these processes have been linked to various diseases, especially in the cardiovascular system. Although cardiomyocytes generate, and are exposed to, considerable hemodynamic forces that affect their shapes, until recently, we did not know whether cell shape affects gene expression. However, new single-cell trapping strategies, followed by single-cell RNA sequencing, to profile the transcriptomes of individual cardiomyocytes of defined geometrical morphotypes have been developed that are characteristic for either normal or pathological (afterload or preload) conditions. This paper reviews the recent literature with regard to cell shape and the transcriptome and provides an overview of this newly emerging field, which has far-reaching implications for both biology, disease, and possibly therapy.

## Introduction

Cells are constantly exposed to mechanical stress by forces that are generated by gravity, organ motion, blood flow, extracellular cell-to-cell and cell-to-matrix interactions, and intracellular traction. These mechanical forces have been shown to influence a broad spectrum of cellular behaviors, from proliferation and differentiation to transcriptional responses and gene expression.

Mechanotransduction is a fundamental biological process, by which cells sense mechanical stimuli, integrate them, and convert them into biochemical signals, inducing downstream cellular responses. Because biomechanical force can be transmitted through the cytoskeleton, microtubules, and actin stress fibers, its propagation time is much shorter than the diffusion of molecules in signaling pathways. This means that the cells respond rapidly to their dynamic environment. Defective mechanotransduction has been associated with numerous diseases from all fields of medicine, including cardiology, dermatology, gastroenterology, nephrology, neurology, oncology, ophthalmology, orthopedics, pediatrics, pulmonary medicine, reproductive medicine, and urology. Abnormal mechanotransduction can be caused by alterations in, or malfunctions of, one or more of the entities involved in the force sensing and conversion process, namely, the extracellular matrix (ECM), cell surface receptors, the cell cytoskeleton, and the various molecules associated with the signaling cascade that occurs in the cytoplasm or nucleus.

A number of recent studies have shown how the nucleus itself is an important mechanosensing component (Guilluy and Burridge 2015; Guilluy et al. 2014). The mechanical forces exerted on the nucleus envelope could alter chromatin organization, nuclear membrane composition, and gene expression, which then trigger downstream responses (Kirby and Lammerding 2018; Szczesny and Mauck 2017). The LINC complex is a critical component that propagates mechanical forces directly from the cytoskeleton to the nucleus envelope. This complex plays a major role in nuclear shape and position, along with chromatin positioning (Banerjee et al. 2014; Rothballer and Kutay 2013). Moreover, the nuclear lamina connects the nuclear envelope to the chromatin. Both the chromatin and nuclear lamina play a role in the nuclear response to strain (Stephens et al. 2017; Stephens et al. 2018).

## Global cell geometry regulation of cellular processes

Signaling activity at the membranes depends on global cell geometry parameters, such as the cellular aspect ratio (AR), size, membrane surface area, and membrane curvature. In this section, we review key studies that have demonstrated the effects of geometry and cell shape on cellular plasticity, with regard to signaling, metabolic activity, and differentiation.

A paper by Rangamani et al. (2013) has presented a “curvature-dependent mechanism of transient receptor activity enhancement.” The authors showed how the enhancement of kinase activity in downstream signaling pathways could be mediated by transient enhancement of receptor activity, following increased ligand binding in the curved plasma membrane areas of elliptic cells with increasing eccentricity. They demonstrated that information contained in the cell’s shape could be transformed into measurably different MAPK phosphorylation levels in the nucleus. Matter exchange by lateral diffusion, between areas of high and low curvature at the plasma membrane, will eventually equilibrate the ligand-induced inhomogeneity of receptor activity. However, when fission of membrane areas with high curvature occurs, this matter exchange cannot take place. This could lead to stabilization of the enhanced interaction with cytoplasmic effectors at the membrane interface of the endocytosed vesicle.

Cell shape may regulate the lineage commitment of embryonic stem cells. McBeath et al. (2004) showed that the shape of human mesenchymal stem cells (hMSCs) regulated their commitment to osteoblasts or adipocytes, by modulating both the RhoA activity and its effect on ROCK-mediated cytoskeletal tension. There was more ROCK activity in spread cells than that in round cells. Spreading hMSCs have been shown to become osteocytes, whereas round cells evolved into adipocytes.

iPSCs are sensitive to mechanical parameters during the early specification stage. Improvements in myofibril alignment and mechanical output have been achieved with a combination of tunable polyacrylamide substrates and defined geometry of adhesion (Ribeiro et al. 2015). This study showed that hPSC-CMs were engineered to grow in an AR of 7:1 and mimic mature cardiomyocytes (CMs) in terms of electrophysiology and the formation of t-tubules. Moreover, hPSCs-CMs with an AR of 7:1 had higher sarcomere activity and alignment.

Neonatal rat cardiomyocytes (CM) that were plated on substrates that had been patterned to constrain the cells in a specific length:width AR had the best contractile function when the ratios were similar to the cells in a healthy adult heart. In contrast, they performed poorly when the ratios were similar to those of myocytes in failing hearts (Kuo et al. 2012). In the early stages of hypertrophy, cells become wider and this is reflected by an increase in the cross-sectional area, namely, an AR of 1:1. In the later stages of hypertrophy, heart failure (HF) occurs and the cells typically appear elongated, with an AR of 11:1. Therefore, it is not surprising that in vivo models of chronic hypertrophy have reported an increase in left ventricular myocyte length of approximately 30% (Gerdes et al. 1996), while adult CMs treated acutely with hypertrophic stimuli in vitro demonstrated similar increases in cell width instead (Kehat et al. 2011).

Kuo et al. (2012) showed that when CMs were cultured on micropatterned plates, sarcomeres preferentially aligned with the major axis of elongated neonatal CMs. In addition, calcium transient durations were prolonged when the CMs had AR patterns of 11:1, rather than a normal AR. These data highlight that cell shape was a driving factor in calcium metabolism.

CMs grow thicker but retain their normal length, in concentric hypertrophy. On the other hand, dilation of the LV in eccentric hypertrophy has been associated with CM elongation through the sequential addition of sarcomeres. The resulting shape of hypertrophied CMs may, on their own, have an impact on contractile function within the myocardial syncytium. The type of hypertrophic remodeling depends on the trigger and the concomitant progression of contractile dysfunction. For example, concentric hypertrophy is a common response to increased afterload, namely, valve stenosis and hypertensive heart disease, whereas eccentric hypertrophy is often observed when there is ventricular volume overload, due to valve regurgitation or shunts or as a late response to a MI.

Haftbaradaran Esfahani et al. (2019) reported that significantly different gene expression profiles had been detected in CMs with three different ARs: 11:1, 7:1, and 1:1. CMs are clustered, corresponding to their morphotypes, based on the whole genome PCA and t-SNE analyses. While hypertrophy is well known to cause profound changes in gene expression (Kontrogianni-Konstantopoulos et al. 2012), changes that depend on cell shapes at single cell levels have never been reported before.

## Local cell geometry regulation of cellular processes

**Fig. 1:**
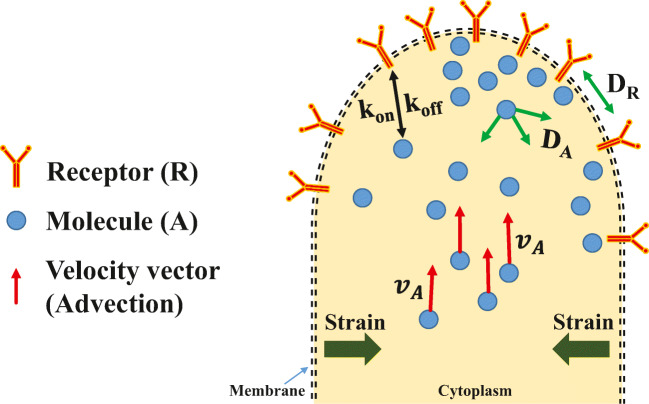
Schematic of reaction-diffusion-advection (RAD) model. External or internal stress causes strain. The membrane receptors that can bind to arbitrary molecules *A* are denoted *R* in the unbound form and *AR* in the bound form. The binding process is $A+R\ \genfrac{}{}{0pt}{}{k_{on}}{\overset{\leftharpoonup }{\overset{\rightharpoonup }{k_{off}}}} AR$. *A* is free to diffuse in any direction, whereas *R* is limited to diffuse along the membrane plane, with the diffusion coefficients of *D*_*A*_ and *D*_*R*_, respectively. The concentration of molecules *A* varies due to diffusion and reaction but also varies due to advection (shown by velocity vector field *v*_*A*_). Advection could be a consequence of strain or cell contraction (e.g., in CMs)

It has been well documented that the effects of global cell geometry influence signaling activity and differentiation, but the local effects are still poorly understood. Cells use various local membrane curves to perform cellular functions, such as filopodia to form adhesions, endosomes for intracellular signaling, and caveolae to moderate membrane tension. Invagination or protrusion of local membranes has been shown to respond differently to stimulus, by increasing cell eccentricity. This is due to the transient assembly of the activated receptors in microdomains with higher curvature. This transient inhomogeneity in the distribution of activated receptors arises from a local imbalance between reactions and the diffusion of soluble ligands and receptors in the plane of the membrane (Rangamani et al. 2013). A schematic of reaction-diffusion-advection system is shown in Fig. 1. This system can be modeled by the partial differential equation for the concentration of substance *X*, which is generally defined as

1 $$ \frac{\partial X}{\partial t}=\nabla .(Xv)+\nabla .\left(D\nabla X\right)+\rho (X) $$

where *v* is the velocity vector field, determining the direction of particle transport due to advection. *D* is the diffusion coefficient and *ρ* accounts for reaction.

When protrusion occurs, there is higher accessibility to external ligands, since the reactive volume to surface ratio is higher for ligand-receptor binding. On the other hand, when invagination occurs, the volume to surface ratio for the ligand-receptor binding is limited, although the release of the intracellular receptor-bound signaling molecule is easier.

Lou et al. (2018) showed that curvature-sensing proteins were involved in how the membrane was manipulated by nanostructures. In addition to membrane curvatures, the authors showed that gene expression was dysregulated after nanostructural deformation of the nuclear envelope.

In another study, Elliott et al. (2015) investigated the closed-loop relationship between regulation of myosin II and fluctuation of local membrane curvature in endothelial cells.

## Cell shape regulates the response to extracellular signals

In addition to the direct role of cell morphology in intracellular signaling, cell shape also regulates the transcriptional responses of the cell to microenvironmental signals.

Cellular geometry influences the cellular response to external biochemical signals, such as cytokines and growth factors. For example, it has been shown that the cellular response to pro-inflammatory TNFα cytokines depends on geometry. One study showed that fibroblasts plated on circular substrate had distinct nuclear translocation of the effector molecule of TNFα signaling, namely, NFκB p65 subunit (Mitra et al. 2017).

The transcriptional responses to external biomechanical and biomechanical signals, such as compressive and external tensile forces, also depend on the geometric state of the cell. Compressive force induces chromatin condensation, leading to reduced transcriptional activity. Since genome spatial organization and transcription factor localization are not similar in cells with different geometries, the transcriptional responses induced by compressive forces also depend on geometry (Damodaran et al. 2018). Both (Damodaran et al. 2018; Mitra et al. 2017) studies compared rectangular and circular cells to investigate the effect of cell geometry on external biochemical and biomechanical signals. However, it is not clear whether the reported differences between both morphotypes were due to their shape, per se. This was because the size of the cells, which was an important confounding factor, was not similar between the two distinct morphotypes. Another study showed that actin-related genes, such as RhoA, Zyxin, and Actin-γ3, were differentially expressed based on the cell size (Jain et al. 2013).

## Cell shape modifies the chromatin and epigenetic machinery

The link between mechanotransduction and epigenetic regulators was the focus of very recent studies published in 2019 and 2020 (Alisafaei et al. 2019; Dreger et al. 2019; Ferrari and Pesce 2019; Mathur et al. 2020). Pereira et al. (2020) reported that the nuclear and cytoplasmic localization of SMYD3 lysine methyltransferase, an epigenetic modulator, was governed by cell shape and AR in murine myoblasts. Moreover, the authors stated that the transcriptional co-activator with PDZ‐binding motif (TAZ) transcription factor, which is an important effector of mechanotransduction pathways (Dupont 2016; Dupont et al. 2011), was regulated by the size of the cell and the AR. In addition, nucleocytoplasmic shuttling of important transcription factors, such as TAZ, YAP, and NFκB, was shown to be modified by cell spreading (Sero et al. 2015). In this context, using three-dimensional microniches in different geometrical features (Bao et al. 2017), it has been observed that cell contractility, nuclear shape, and YAP/TAZ distribution were influenced by cell volume. Interestingly, the mechanosensitivity of chromosomes is also dependent to the orientation of chromosomes inside the nucleus, governed by global geometry of the cell (Wang et al. 2017).

## Cell shape regulates gene expression in HF (cardiomyopathies)

The heart is a mechanosensitive organ that is capable of adjusting force and the rate of contractions to meet mechanical demands. The contractile cells of the myocardium, or CMs, experience different types of mechanical stimuli, including sheer stress and relaxation and contraction cycles. Although it is known that the mechanical environment influences the behavior of CMs, the mechanosensation process remains poorly understood.

Cardiomyopathies, such as hypertrophic cardiomyopathy (HCM) and dilated cardiomyopathy (DCM), are primary disorders of the heart muscle and major causes of HF. They have been associated with high morbidity and mortality. The causes of cardiomyopathies can be environmental, such as infections and exposure to toxins or drugs, or due to genetic mutations. It is believed that changes in the genetic composition of ECM molecules, integrins or cytoskeletal proteins, could be responsible for impaired mechanosensation and various types of cardiac disease.

According to Braunwald (Lilly and Braunwald 2012), the main feature of HCM is an “unexplained hypertrophy of the left ventricle (and sometimes of the right ventricle), often with predominant involvement of the interventricular septum.” It is also characterized by diastolic dysfunction and myocyte disarray and fibrosis. In most cases, the contractile apparatus of the heart is affected by mutations in sarcomeric proteins, leading to increased contractility of the myocytes. DCM is characterized by dilatation of one, or both, ventricle and has a familial etiology in 30 to 50% of cases. It affects a wide range of cellular functions, leading to impaired contraction of the myocytes, cell death, and fibrotic repair.

Mechanosensation, which is the perception of mechanical stimuli, and mechanotransduction, which is the conversion of mechanical stimuli into informative cellular signals, are cell membrane-dependent, fundamental processes in biology (Gillespie and Walker 2001; Kung 2005). In the cardiovascular system, CMs generate, and respond to, various types of biomechanical stress in order to maintain continuous contractile function of the heart. This, in turn, alters their cellular architecture (Garoffolo and Pesce 2019).

Different types of cardiac hypertrophy have been associated with increased cell volumes, defined as cellular hypertrophy, and changes in cell shape. While the effects of cell volume on gene expression are well known (Knoll et al. 2011), the effects of cell shape on gene expression are not so well understood. CMs are dynamic cells that have the ability to change their shape during mechanically induced remodeling processes, and this affects their contractile functions and causes aberrations in cellular calcium metabolism. This has led us to hypothesize that cell shape could have a “global” impact on the transcriptome. Furthermore, the cell membranes in the heart are constantly reshaped by the cycles of contraction and relaxation. This contraction causes sarcolemma, between two neighboring z-disks, to bulge out and flatten in each cycle of contraction and resting. Accordingly, we developed the hypothesis that “local” and microdomain effects of membrane curvature could be of the highest importance for cardiac myocyte plasticity and signaling. This effect may depend on the frequency of the contraction. These novel concepts may also help to explain how changes in frequency, and thus membrane shape, affect cardiac function (Knoll 2015).

A study by Haftbaradaran Esfahani et al. (2019) showed that the number of detected genes was significantly lower when the ARs were 1:1 and 11:1, compared with an AR of 7:1. Moreover, that study showed that ARs of 1:1 and 11:1 led to downregulation of the investigated genes in comparison with an AR of 7:1. The shape-dependent regulation of the number of expressed genes, in response to deviation from the normal cell shape, was an entirely new finding, and it led to a downregulation in that study. This result may have implications for different types of HF, where CMs undergo characteristic changes in cell shape. This may, in turn, lead to silencing of specific genes.

In line with the significant downregulation of gene expression in ARs of 1:1 and 11:1, the majority of canonical pathways in both ARs are also downregulated. Intriguingly, hypertrophic markers generally display different responses for eccentric and concentric morphotypes, indicating that the cellular responses follow different strategies. These depend on the lateral or radial polarity of the cell. Clearly, dissimilar regulatory pathways are activated by different shapes.

## Summary and outlook

**Fig. 2:**
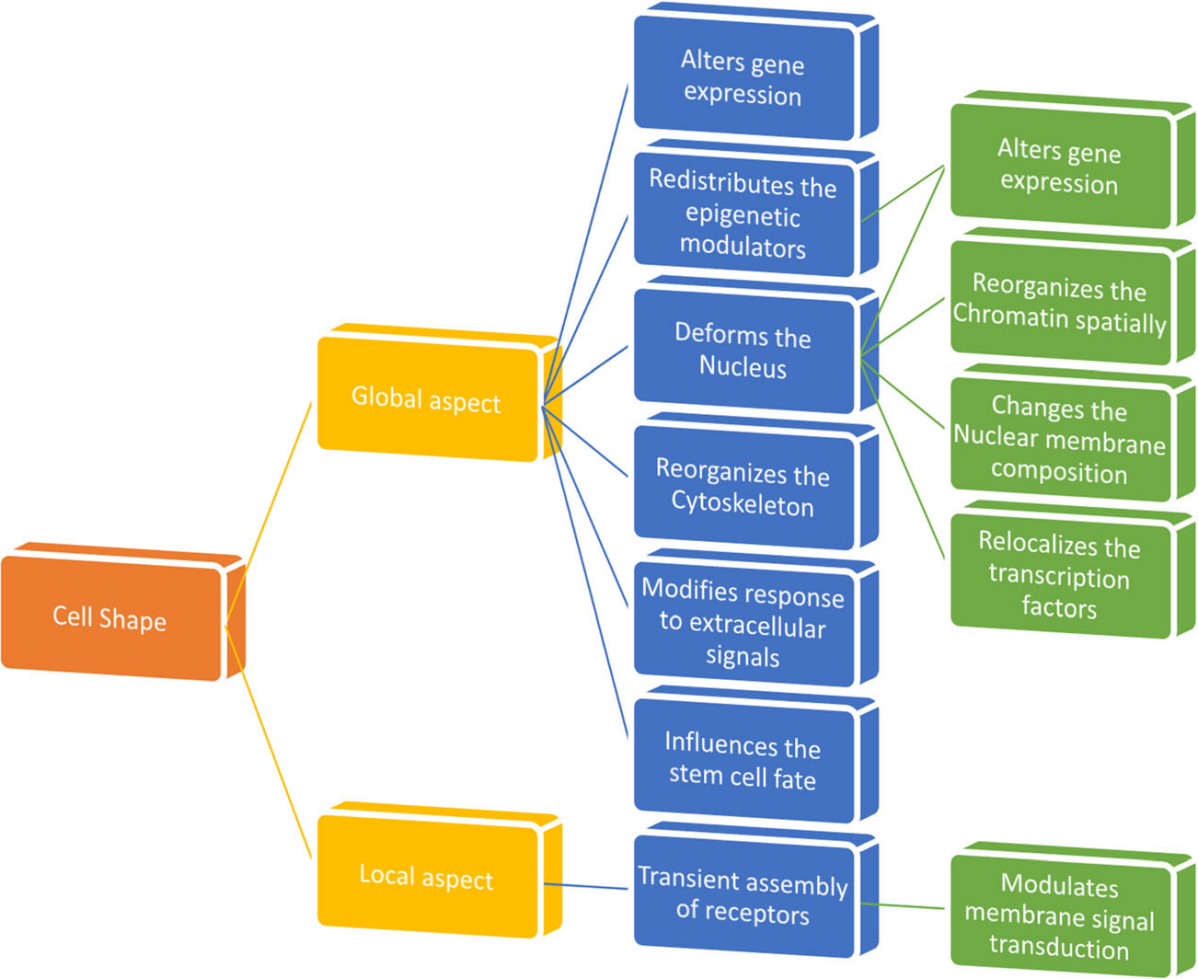
Any changes in cell shape, either globally (such as size, volume, AR, or geometry) or locally (such as protrusion or invagination of local membranes), cause substantial consequences from modifying cellular architecture to altering gene expression

Any changes in cell shape, either globally (such as either size, volume, AR, or geometry) or locally (such as protrusion or invagination of local membranes), cause substantial consequences from modifying cellular architecture in altering gene expression (Fig. 2). The novel observations that cell shape has a powerful effect on gene expression mean that we could potentially manipulate the genome, which could have far-reaching implications for biology and medicine.

In addition, it would be desirable to analyze the effects of cell shape on other types of RNA gene expression, for example, in terms of cell shape effects on micro-RNA and large noncoding RNA (lncRNA), among others. Moreover, the effects of cell shape on gene expression should be analyzed using much shorter time periods, such as seconds, minutes, hours, and not just after a few days. Likewise, the effects of cell shape on gene expression should be analyzed using much longer follow-up periods, such as weeks, months, or even years. This is probably not technically possible at the moment, but it is likely to be in future. In addition, it is also important to find out to what extent the proteome, metabolome, and epigenome undergo specific changes following alterations in cell shape. Therefore, it is crucial to develop novel assays for these different experimental approaches, especially when it comes to simultaneous analysis in single cells.

**Fig. 3:**
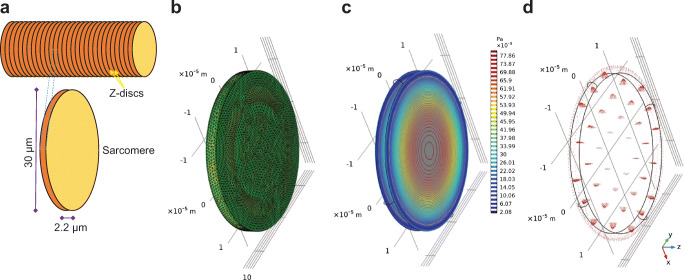
A COMSOL program was designed to mathematically simulate the advection of an arbitrary molecule *A*, floating in one sarcomere of a CM towards the sarcolemma. The advection is due to contraction of CM. For a CM to contract, the sarcomere must shorten from 2.2 to 1.6 μm. **a** One CM was assumed as a cylinder, consisting of several symmetric sarcomeres. Since sarcomeres are similar repeating units, one contracting sarcomere was modeled. Moreover, it was assumed that the liquid inside the sarcomere is incompressible. **b** The finite-element mesh was generated to divide the model into small elements, over which a set of Navier-Stokes equations were solved. **c** When the sarcomere contracts, the pressure from the liquid is exerted to the contracting z-disks. The exerted pressure in different parts of the z-disk is shown by color-coded contours. **d** The arrows are the velocity vector (advection), caused by shortening the sarcomere. The arrow length is proportional to the magnitude of velocity

Comprehensive tissue-scale models are critical for predicting geometry-dependent cellular signaling. These are based on platforms that have been specifically designed for modeling cell biological systems, such as virtual cell (VCell) software or general multi-physics software like COMSOL Multiphysics. To illustrate this point, we designed a COMSOL program to mathematically simulate the advection of an arbitrary molecule, floating in one sarcomere of a CM towards the sarcolemma (Fig. 3). In this model, the advection was due to the contraction of the CM. Navier-Stokes equations were used to calculate the liquid flow. This showed that the liquid flow, depended on the dynamic geometry of the sarcomere, can be estimated by simulation.

Last but not least, it will probably be essential to find out to what extent cardiac pathologies can be mimicked by different ARs. We also need to know to what extent aberrations in the genetic code, namely, mutations, affect the effects of cell shape on cellular signal transduction and overall information processing. Other important aspects include identifying the effects of cell shape on other cell types, such as fibroblasts or endothelial cells, their interplay, and to what extent cell shapes can be used to counteract the effects of various pathologies, including mutations in genes that cause diseases.
